# High Gamma Activity in the Infralimbic Cortex to Nucleus Accumbens Shell Pathway Modulates Innate Aversion Differentially across Sex

**DOI:** 10.1523/ENEURO.0297-25.2025

**Published:** 2025-09-09

**Authors:** Elijah C. Grablin, Joaquin E. Douton, Regina M. Carelli

**Affiliations:** Department of Psychology and Neuroscience, The University of North Carolina, Chapel Hill, North Carolina 27599

**Keywords:** hedonics, optogenetics, oscillations, quinine, rats, taste

## Abstract

Aversion modulation is a key component of hedonic processing, and its dysfunction is evident in psychiatric illnesses. The infralimbic cortex (IL) to nucleus accumbens shell (NAcSh) pathway is essential in hedonic processing in rodents but operates differentially across sex, with beta (20 Hz) oscillatory activity involved in learned aversion in male but not female rats. In this study, we used taste reactivity (TR) and electrophysiology to examine the role of high gamma (80 Hz) activity in affect modulation, specifically innate (quinine) and learned (conditioned taste aversion, CTA) aversion, in male and female Sprague Dawley rats. Local field potential (LFP) recordings in males showed no changes in IL or NAcSh activity, or in IL→NAcSh functional connectivity, in the high gamma frequency band during innate or learned aversion. In contrast, in females, quinine elicited an increase in IL and NAcSh 80 Hz LFP activity and IL→NAcSh functional connectivity. Interestingly, LFP directionality analyses in females indicated that top-down modulation from IL to NAcSh was associated with innate aversive behavior expression. To confirm a causal link of 80 Hz activity in aversion processing in females, optogenetics was used. Here, optogenetic stimulation of the IL→NAcSh pathway did not affect learned (CTA) aversion but it selectively decreased innate (quinine) aversion. Collectively, these results highlight sex- and frequency-specific differences in aversion modulation by the IL→NAcSh pathway, with high gamma frequencies involved in modulating innate aversion, specifically in female rats.

## Significance Statement

Accurate regulation of aversion is essential for healthy emotional processing and its disruption has been linked to several psychiatric illnesses. The infralimbic cortex to nucleus accumbens shell pathway is key for hedonic processing, but its function differs across sex. Here, we assessed how “high gamma” brain waves within this pathway encodes innate and learned aversion in both male and female rats. Using local field potential recordings, we showed that high gamma frequencies specifically modulate innate aversion in female but not male rats, with causal links in females confirmed by optogenetics. These findings highlight sex- and frequency-specific differences in hedonic processing that could have implications for developing effective, sex-specific treatments for mental health disorders.

## Introduction

Hedonic processing is critical for guiding adaptive behaviors and is often disrupted in psychiatric illnesses (Davey, 2011; Anderson et al., 2021; Tolchinsky et al., 2024). To study hedonic processing in rats, we use taste reactivity (TR) in which solutions of different hedonic valence are infused directly into the oral cavity and orofacial responses are recorded and analyzed to infer affective states (Grill and Norgren, 1978a,b). Here, sweet solutions (e.g., saccharin) elicit appetitive TR associated with the ingestion of the liquid, and bitter solutions (e.g., quinine) elicit aversive TR, usually linked to the expulsion of the liquid from the mouth. Importantly, TR can be modulated through learning via conditioned taste aversion (CTA), where an innately rewarding solution is paired with the injection of a malaise-producing agent (e.g., lithium chloride) resulting in a shift in TR from appetitive to aversive. Thus, TR reveals the affective state of the rat and how this state can change with learning.

Our lab has examined how the brain processes innate and learned aversion using TR and CTA (Roitman et al., 2005, 2008; Wheeler and Carelli, 2008; Saddoris et al., 2014; Douton and Carelli, 2023). For example, we showed that nucleus accumbens shell (NAcSh) neurons exhibit different firing profiles to rewarding and innately aversive tastants (Roitman et al., 2005, 2008; Wheeler and Carelli, 2008). Further, the NAcSh tracks the valence of the tastant, as CTA can shift the firing pattern from an inhibitory (rewarding-like) to an excitatory (aversive-like) profile (Roitman et al., 2005, 2008; Wheeler and Carelli, 2008).

The NAcSh receives dense projections from the infralimbic cortex (IL), the rat homolog of the human ventromedial prefrontal cortex (Eisenberger et al., 2003; Phan et al., 2005), which has been implicated in learned aversion (Sesack et al., 1989; Takagishi and Chiba, 1991; Reynolds and Berridge, 2002). For example, the IL tracks the hedonic valence of different taste solutions, and CTA produces a similar shift in IL activity from a rewarding-like to an aversive-like firing profile (Hurley et al., 2023). Interestingly, beta frequencies are involved in CTA modulation, as 20 Hz optical activation of the IL→NAcSh pathway reduced aversive TR during CTA, but only in male rats (Hurley and Carelli, 2020). Likewise, local field potential (LFP) recordings revealed differential effects of CTA on IL and NAcSh 20 Hz oscillatory activity in male and female rats (Douton and Carelli, 2023), supporting differential roles of beta frequencies in the IL→NAcSh pathway in learned aversion across sex.

High gamma frequencies (65–100 Hz) have been associated with reward processing, decision-making, and behavioral flexibility (Berke, 2009; Van Der Meer, 2009; West et al., 2021). While high gamma activity has also been linked to some forms of aversion (Dejean et al., 2016; Tan et al., 2019), those investigations examined learned aversion in brain regions other than the IL and NAcSh. Notably, we identified significant sex differences in oscillatory signaling dynamics within the IL and NAcSh during quinine infusion, specifically within the 70–100 Hz high gamma frequency band (Douton and Carelli, 2023). However, variations in high gamma oscillatory activity and its causal links to both innate and acquired aversion in either sex is largely unexplored.

Given these findings, we used TR and electrophysiological (LFP) methods to examine how high gamma frequencies in the IL→NAcSh pathway respond to tastants of different hedonic valence (neutral, rewarding, conditioned-aversive, and innate-aversive) to reveal the functional role of high gamma activity in innate and learned aversion across sex. Results showed an increase in power, functional connectivity, and a top-down directionality during quinine infusions (innate aversion) in female but not male rats. To confirm causality of the IL→NAcSh pathway in aversive processing in females, we applied 80 Hz optogenetic activation of glutamatergic projections from the IL to the NAcSh during innate or learned (CTA) aversion. Collectively, our findings indicate that at the high gamma frequency range, the IL→NAcSh pathway plays an important role in modulating innate but not learned aversion, specifically in female rats.

## Materials and Methods

### Animals

Sprague Dawley (*n* = 41) rats weighing between 220 and 350 g at the beginning of the study were used (Inotiv). Rats had *ad libitum* access to food and water except during behavioral testing where they were water-restricted to 16–20 dl/day. Rats were single housed, maintained on a 12 h reverse light/dark cycle, and completed behavioral testing during the dark phase. All protocols were conducted in accordance with the National Institutes of Health Guidelines for the Care and Use of Laboratory Animals and approved by the University of North Carolina at Chapel Hill Institutional Animal Care and Use Committee. This manuscript includes additional analyses of data that partially overlap with those reported in a previous publication (Douton and Carelli, 2023), providing new insights not presented in the original study.

### Apparatus

This study was conducted in experimental chambers similar to those described in detail previously (Douton and Carelli, 2023). Briefly, operant chambers (43 × 43× 53 cm) with a transparent plexiglass floor (Med Associates) were housed within a sound-attenuating cubicle. A GoPro camera (GoPro) was positioned to face a mirror below the chamber to record oromotor responses to intraoral infusions. Intraoral infusions were delivered via Tygon tubing that was connected to a blunted needle and attached to a syringe on a pump. Both the laser stimulation and IO infusions were controlled by MED-OC software (Med Associates). Boxes used for electrophysiological recording had 1× gain headstages connected via a flexible recording cable to a commutator (Crist Instrument) and then to a multichannel acquisition processor (MAP) system (Plexon).

### Electrode array implant

For the electrophysiology experiment, rats (*n* = 23) were anesthetized with a mixture of ketamine (100 mg/kg) and xylazine (10 mg/kg, intraperitoneal) and implanted with an intraoral cannula, as above. In addition, electrode arrays containing eight 50 µm stainless steel, Teflon-coated microwires (NB Labs) were implanted in the IL (+2.8 AP, +0.5 ML, −5.0 DV) and the NAc shell (+2.0 AP, +2.1 ML, −6.3 DV at a 10 °angle) as described previously (Wheeler et al., 2008; West et al., 2014). For each of the electrode arrays, a ground wire was wrapped around a screw and implanted into the brain. Screws and arrays were secured into place using dental acrylic (Lang Dental). Animals were given 1 week of recovery with *ad libitum* food and water. In addition, rats were given subcutaneous injections of meloxicam (1 mg/kg/day for 3 d), and, to aid in food consumption, wet food was given for a week following the implantation of the IO cannula.

### Electrophysiology experiment

**Figure 1.**
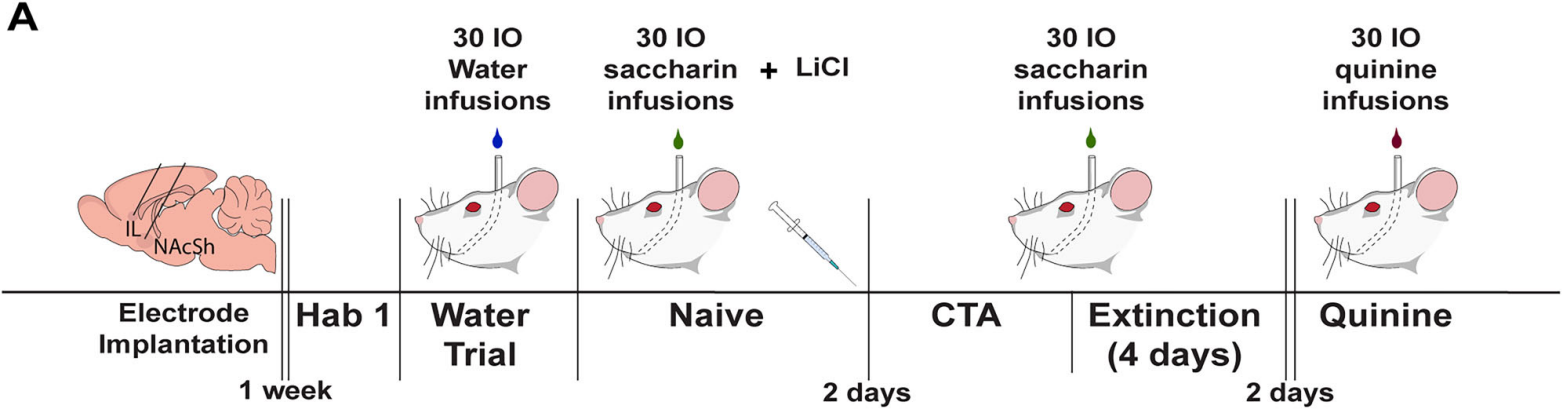
Schematic timeline: electrophysiology experiment. Rats had electrode arrays implanted into the IL and the NAcSh, given 1 week to recover, then habituated to IO water infusions. Rats then received IO infusions of saccharin immediately followed by an injection of LiCl. After a 2 d break, the CTA session was completed. Following 4 extinction days, rats were given IO infusions of quinine. LFP activity was monitored for 15 min before the start of and during each session, except during habituation. TR was measured throughout IO infusions. Hab, habituation; CTA, conditioned taste aversion.

A timeline of the electrophysiology experiment is illustrated in Figure 1. After recovery from surgery (1 week), rats (*n* = 15) were water deprived (15 ml/day) and habituated to the experimental chamber and IO water infusions for 2 d. The second day of habituation, which included IO water infusion, was used to assess electrophysiological activity in response to water in some females (*n* = 5). Next, female (*n* = 6) and male (*n* = 9) rats underwent the CTA procedure as described previously (Douton and Carelli, 2023). Briefly, rats received IO saccharin infusions followed by LiCl injection (naive). Two days later, they were re-exposed to saccharin (CTA). After 4 d of extinction (saccharin infusion only), rats were exposed to IO quinine infusions. Electrophysiological activity was recorded for 15 min prior to the start of each session and during the 30 IO infusions of the tastants. A second experiment was conducted to assess electrophysiological activity in response to water in males. Briefly, water-deprived (15 ml/day) male rats (*n* = 8) were habituated to the experimental chamber and IO water infusion for 1 d. On the following day, electrophysiological recordings were obtained during a second day of water infusion [30 infusions, 200 µl per infusion on a variable time (VT) 30 s schedule]. All behaviors were recorded using a GoPro camera for future video analysis of taste reactivity (TR). Male and female data in response to saccharin, CTA, and quinine infusions represent additional analyses of previously published data (Douton and Carelli, 2023).

### Electrophysiology (LFP) recordings

LFPs were acquired at 1 kHz using commercially available neurophysiological systems (OmniPlex System; Plexon) described previously (Douton and Carelli, 2023). Briefly, raw electrical outputs from an individual wire implanted within either the IL or NAcSh were low-pass filtered (200 Hz). LFP power spectra were computed using fast Fourier transform of 2,048 points and exported from NeuroExplorer. Perievent spectrograms were generated using a *z*-score calculation of event-related spectral perturbations (ERSPs; 0.2 ms bins) between 75 and 85 Hz frequency band. The *z*-score was calculated as: (powerinbin−averagebaseline/SDbaseline). The spectrogram was normalized to baseline (2 s prior IO delivery), and the average *z*-score during the 2 s baseline and the 3.5 sec IO delivery were graphed for each tastant. For visualization, maximum and minimum values were set at two standard deviations from baseline (*z* = ±2).

#### Behavioral analysis

TR was analyzed as total number of appetitive and aversive responses based on prior work (Douton and Carelli, 2023; Hurley et al., 2023). Briefly, lateral tongue protrusions and rhythmic tongue protrusions were classified as appetitive, and gaping behavior was categorized as aversive. Neutral responses were characterized as low amplitude, bilaterally symmetric movements of the mouth (Grill and Norgren, 1978b). TR measurement was conducted by an experimenter blind to the experimental conditions. Differences in TR between groups were analyzed using a *t* test with a Welsh's correction. A two-way repeated-measures ANOVA was used to examine group differences during the analysis of TR over the course of the session.

### Electrophysiology coherence analysis

To analyze coherence between the IL and NAcSh, raw signals for each individual session were exported into Matlab, and the function “*mscohere*” was used with Hann window, 50% overlap and a nonuniform fast Fourier transform (NFFT) of 2,048. Then, like the perievent spectrogram, a perievent coherogram (coherence change relative to baseline across time) between 75 and 85 Hz was built by calculating a *z*-score using 2 s prior to infusion as a baseline. An infusion was considered to increase coherence if, during the 3.5 s IO infusion duration, there was an increase of ≥2 SD relative to baseline (*z* = 2) in the group average coherogram. An infusion was considered to reduce coherence if a reduction ≥2 SD relative to baseline (*z* ≤ −2) was observed. An infusion was considered to produce both if an increase and a decrease in coherence relative to baseline was observed during the 3.5 s. If no change greater than 2 SD relative to baseline was observed, it was considered as no change in coherence.

### Electrophysiology directionality analysis

IL and NAcSh lead was calculated by performing IL→NAcSh power-power cross-correlation iteratively across 1 s and moving by 5 ms throughout the 3.5 s infusion duration as described previously (Adhikari et al., 2010; Likhtik et al., 2013). Briefly, signals for each infusion bin were averaged and then band-passed filtered between 78 and 82 Hz using the Matlab finite impulse response filter function “*fir1*” with a sampling frequency order and a Hamming window. Then, the instantaneous amplitude for each signal was extracted using the Hilbert transform (Matlab function “*hilbert*”), and the mean amplitude for each signal was removed. Then, the Matlab “*xcorr*” function was used to calculate lags ranging from −100 to 100 ms, and the lag at which the cross-correlation peaked was calculated for each of the windows and then averaged for each rat.

### Electrophysiology data analysis

Changes in *z*-scored power were analyzed relative to baseline using a paired *t* test. Comparison of proportion of sessions was performed using χ^2^ tests. For lag analysis, each session was divided into four bins: Bin 1, IO1–7; Bin 2, 8–14; Bin 3, 15–22; Bin 4, 23–30. Then, lags for each session were analyzed using Wilcoxon's two-tailed singed-rank nonparametric test to test whether the mean distribution during each bin was significantly different from 0. Lags were also correlated with gaping behavior using Pearson's correlation. All analyses were completed in GraphPad prism version 10.3.1 and *p* < 0.05 was considered significant. Of note, recordings for one of the female rats was not reliable during water infusion and not used.

### Optogenetic surgeries

Surgeries were performed as described previously (Hurley and Carelli, 2020). To induce virus expression in glutamatergic IL neurons, rats were anesthetized with a mixture of ketamine (100 mg/kg) and xylazine (10 mg/kg, intraperitoneal) and microinjected with 500 nl of mCherry (AAV5-CamKIIa-mCherry; UNC Vector Core; *n* = 13) or channelrhodopsin, ChR2 [AAV5-CamKIIa-hchR2(h134R)-mCherry, *n* = 14] (RRID:SCR_008759) under the CAMKII promoter bilaterally in the IL (coordinates: +2.7 mm anteroposterior, ±0.5 mm medial-lateral, 4.7 mm dorsal-ventral from the skull) at a concentration of 3.2 × 10^12^ viral genomes/ml. Virus was microinjected at a rate of 100 nl/min, and injectors were left in place for 8 min postmicroinjection to ensure virus spread. Importantly, our lab (West et al., 2021) and others (Zhao et al., 2011; Ting and Feng, 2013) have shown that ChR2 H134R can sustain light-evoked firing at 80 Hz with high fidelity. Rats were allowed at least 6 weeks of recovery for virus to express in IL terminals in the NAcSh. A second surgery was performed to implant an intraoral (IO) cannula and optical fibers (200 µm core) aimed at the medial rostral NAcSh (+1.5 mm anteroposterior, ±2.1 mm medial-lateral, −6.5 mm dorsal-ventral from skull, lowered at a 10° angle), using established procedures (Roitman et al., 2005; Wheeler et al., 2008; Hurley et al., 2017; Hurley and Carelli, 2020).

### Optogenetic experiment

A timeline of the optogenetic experiment is illustrated in Figure 5A. Female rats were divided into two treatment groups: mCherry (*n* = 9) and ChR2 (*n* = 9). After viral or mCherry injection, optical fiber and intraoral cannula implantations, and recovery (1 week), rats were habituated to the operant chamber (2 d), mildly water restricted (15 ml/day), and then habituated to IO infusions (IO water delivered, 1 d). The following day (naive), rats received 30 IO infusions (200 µl over 3.5 s/infusion on VT30 sec schedule) of 0.15% saccharin followed by an injection of lithium chloride (127 mg/kg, i.p.) to induce CTA. Following 2 d of recovery, rats were placed back in the chamber and underwent CTA test (CTA). Here, to test the effects of optical stimulation on CTA, rats were re-exposed to the 30 IO saccharin infusions paired with 80 Hz optogenetic stimulation of the NAcSh (3.5 s duration, 10 mW 473 λ laser). Finally, following 4 d of IO saccharin infusions only (extinction), rats received 30 IO infusions of quinine (1 mM) paired with 80 Hz optogenetic stimulation (quinine). The orofacial behaviors of each rat were recorded using a GoPro camera for future video analysis of TR and behavior was analyzed, as described above. One rat was removed from the CTA phase and another rat was removed from the quinine phase due to IO leakage.

### Histology

After testing, concluded rats were deeply anesthetized with a mixture of ketamine (100 mg/kg) and xylazine (10 mg/kg, i.p.). To mark the placement of electrode tips in the electrophysiology experiment, following anesthesia, rats received a current (13.5 µA) passed through each microwire electrode for 5 s. Afterward, brains were extracted and fixed in 20% sucrose, 3% potassium ferrocyanide, and 10% buffered formalin. After postfixing and freezing, 40 µm coronal brain sections were sliced and mounted on slides. The inclusion of potassium ferrocyanide facilitated a blue reaction at the electrode tip location, which was visualized under a wide-field microscope objective. The precise placement of the electrode tip within the infralimbic cortex (IL) or nucleus accumbens shell (NAcSh) was determined by examining the relative position of the visible reaction product in relation to anatomical landmarks and the stereotaxic atlas (Paxinos and Watson, 2006). Only data from electrodes with histologically confirmed locations in the IL and NAcSh were used in this analysis. Rats in the optogenetic experiment were perfused transcardially with phosphate-buffered saline followed by 4% paraformaldehyde (MilliporeSigma). The brains were collected, postfixed in 4% paraformaldehyde for 24 h followed by 30% sucrose in phosphate buffer, and then sectioned. Slices were mounted on slides and imaged using a LEICA fluorescence microscope. Only rats with viral expression in both the IL and the NAcSh and optical fiber placement in the NAcSh were included.

## Results

### High gamma LFP activity in the IL and NAcSh is not involved in encoding salient taste stimuli or aversive stimuli in male rats

**Figure 2.**
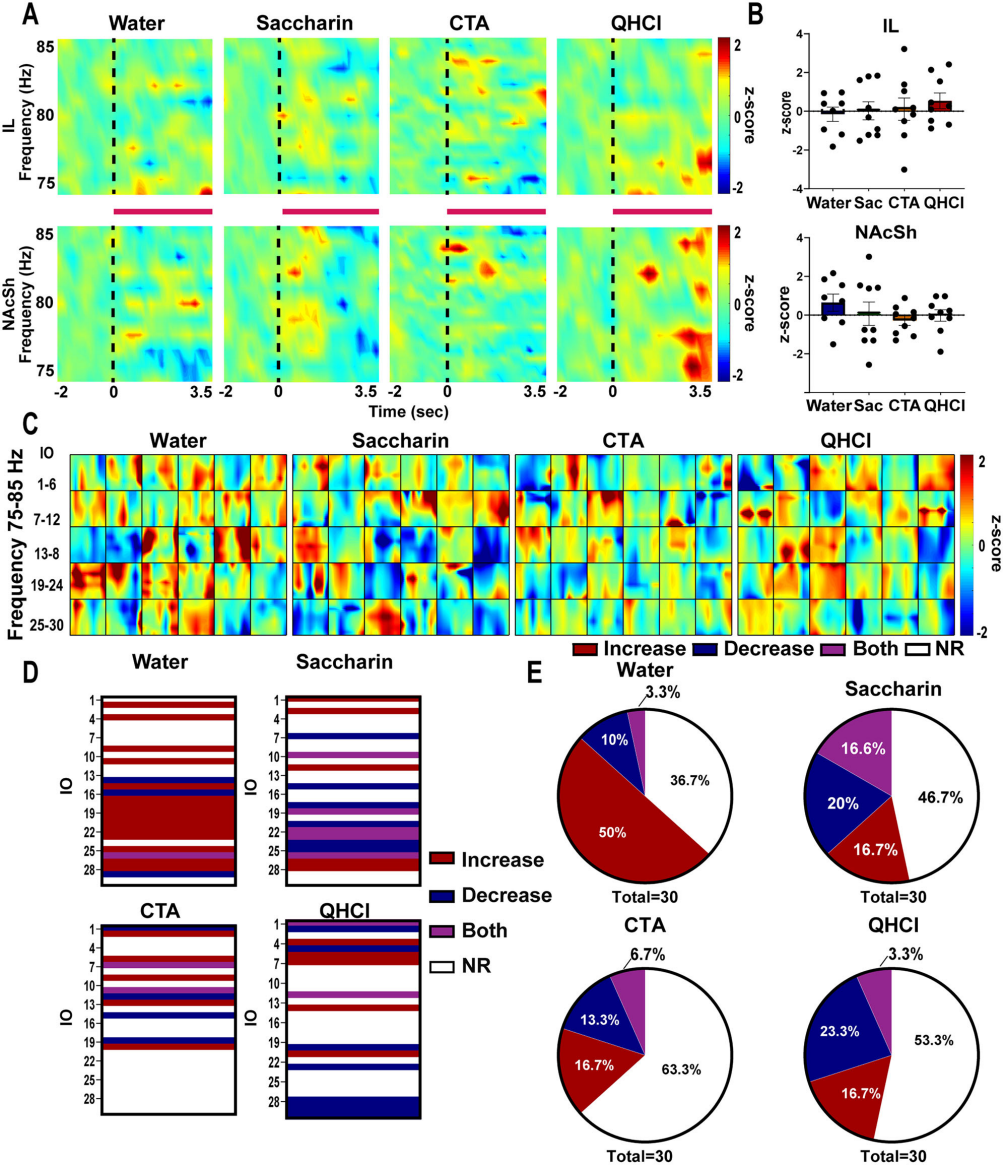
High gamma activity in the IL and NAcSh is not involved in encoding salient taste stimuli or aversive stimuli in male rats. ***A***, Average perievent spectrograms (*z*-score) showing changes in oscillatory activity after IO infusions (onset of infusion represented by dotted black line) during water (left), saccharin (middle-left), CTA (middle-right), and quinine (right) sessions in both the IL (top) and NAcSh (bottom). *Z*-score relative to baseline is shown on overlay (right). ***B***, Bar graphs showing the mean *z*-score at 80 Hz for each rat during water (blue), saccharin (Sac -green), CTA (orange), and quinine (red) sessions in the IL (top) and the NAcSh (bottom). ***C***, Average coherograms (*z*-score) during 3.5 s infusion duration in response to each of the 30 infusions during water (left), saccharin (middle-left), CTA (middle-right), and quinine (right). *Z*-score relative to baseline is shown on overlay. *z* ≥ 2 were considered increase (red) in coherence, *z* ≤ 2 were considered decrease (blue) in coherence and −2 ≤ *z* ≤ 2 was considered no change in coherence. ***D***, Categorization of each infusion (IO) of water (top left), saccharin (top right), CTA (bottom left), and quinine (bottom right) according to its coherence response. Each infusion elicited either an increase (red), decrease (blue), both (purple), or no change (NC-white) in coherence. ***E***, Pie charts showing the percentage of infusions that elicited an increase (red), decrease (blue), both (purple), or no change (NC-white) in coherence for water (top left), saccharin (top right), CTA (bottom left), and quinine (bottom right). QHCl, quinine; CTA, conditioned taste aversion; IL, infralimbic cortex; NAcSh, nucleus accumbens shell.

First, we performed time–frequency analyses of in vivo electrophysiological LFPs to understand how oscillatory activity at high gamma frequency in the IL and the NAcSh varies in response to taste stimuli of different hedonic valence in male rats. Figure 2*A* depicts perievent spectrograms showing average changes in LFP oscillatory activity in response to the IO infusions (red lines) of water (left), saccharin (middle-left), saccharin following CTA (middle-right), or QHCl (right) relative to the 2 s baseline period prior to the onset of each infusion (black dotted lines). Neither neutral, rewarding, nor conditioned-aversive stimuli (CTA) elicited changes in 80 Hz activity in either region. For quinine, red zones within the spectrogram show a slight activation toward the end of the infusion, particularly in the NAcSh; however, this change was observed primarily at frequencies lower than 80 Hz. Bar graphs in Figure 2*B* summarize average activity across rats for each tastant within the IL (top) and NAcSh (bottom) at 80 Hz; again, no significant changes in neural activity were observed during the IO infusions relative to baseline in either region (IL: water: *t*_(7)_ = 0.3947, *p* = 0.3704; saccharin: *t*_(8)_ = 0.0785, *p* = 0.939; CTA: *t*_(8)_ = 0.1714, *p* = 0.868; QHCl: *t*_(8)_ = 1.348, *p* = 0.214; NAcSh: water: *t*_(7)_ = 1.585, *p* = 0.156; saccharin: *t*_(8)_ = 0.052, *p* = 0.959; CTA: *t*_(8)_ = 1.188, *p* = 0.268; QHCl: *t*_(8)_ = 0.0486, *p* = 0.962).

Next, to assess how these taste stimuli affect IL→NAcSh functional connectivity at the high gamma frequency band, we analyzed the average change in IL→NAcSh coherence across infusions for each tastant. Figure 2*C* shows average coherograms (change in coherence relative to baseline across time) in response to each of the 30 IO infusions of water (left), saccharin (middle-left), CTA (middle-right), and QHCl (right). Each box represents IL→NAcSh coherence change (*z*-scored) during the 3.5 s period of each infusion, averaged across rats. We defined that an infusion increased coherence if it elicited an increase ≥2 SD (*z* ≥ 2; red) from baseline (2 s prior onset of infusion), decreased coherence if it elicited a reduction ≥2 SD (*z* ≤ −2; blue) from baseline (2 s prior onset of infusion), both if it elicited both responses, or no change (NC) if it did not change coherence. Figure 2*D* shows how coherence within each infusion across all rats was categorized with increased coherence (red), reduced coherence (blue), simultaneous increase and decrease (purple), and no change (white). In males, we observe that water mainly elicited an increase in coherence, specifically during the second half of the session (IOs 16–30). In contrast, saccharin in the naive state, saccharin following CTA, and quinine infusions elicited mainly no change in coherence or both increase and decrease during the same infusion. To compare these effects, the pie charts in Figure 2*E* illustrate the proportion of each type of response across tastants. As mentioned, in males, when the taste stimulus was neutral (i.e., water), most of the IO infusions showed an increase in coherence (50%), while 36.7% showed no change, 3.3% of infusions elicited simultaneous increases and decreases, and only 10% showed reduced coherence. Remarkably, when the taste stimulus was salient (i.e., either appetitive for naive saccharin, conditioned in CTA, or innately aversive), most of the infusions did not elicit a change in coherence (saccharin: 46.7%; CTA: 63.3%; QHCl: 53.3%), and the rest of infusions were divided almost equally between reduced coherence (saccharin: 20%, CTA: 13.3%; QHCl:23.3%), simultaneous increase and decrease (saccharin: 16.6%, CTA: 6.7%, QHCl: 3.3%), or increased coherence (16.7% for saccharin, CTA, and QHCl). Consequently, the profile elicited by water was significantly different from saccharin (χ^2^_(2,60)_ = 9.027, *p* = 0.0289) and QHCl (χ^2^_(2,60)_ = 7.859; *p* = 0.049), trending between water and CTA (χ^2^_(2,60)_ = 7.610, *p* = 0.0548), but not different between saccharin and CTA (χ^2^_(2,60)_ = 2.443, *p* = 0.4856), saccharin and QHCl (χ^2^_(2,60)_ = 1.496, *p* = 0.6832), or CTA and QHCl (χ^2^_(2,60)_ = 1.075, *p* = 0.783). Collectively, these results indicate that while neither neutral, rewarding, nor aversive taste stimuli elicit significant activity in either the IL or the NAcSh at 80 Hz, there is an increase in functional connectivity between the IL and NAcSh during the infusion of water in males.

### High gamma LFP activity in the IL and NAcSh is involved in encoding quinine aversion in female rats

**Figure 3.**
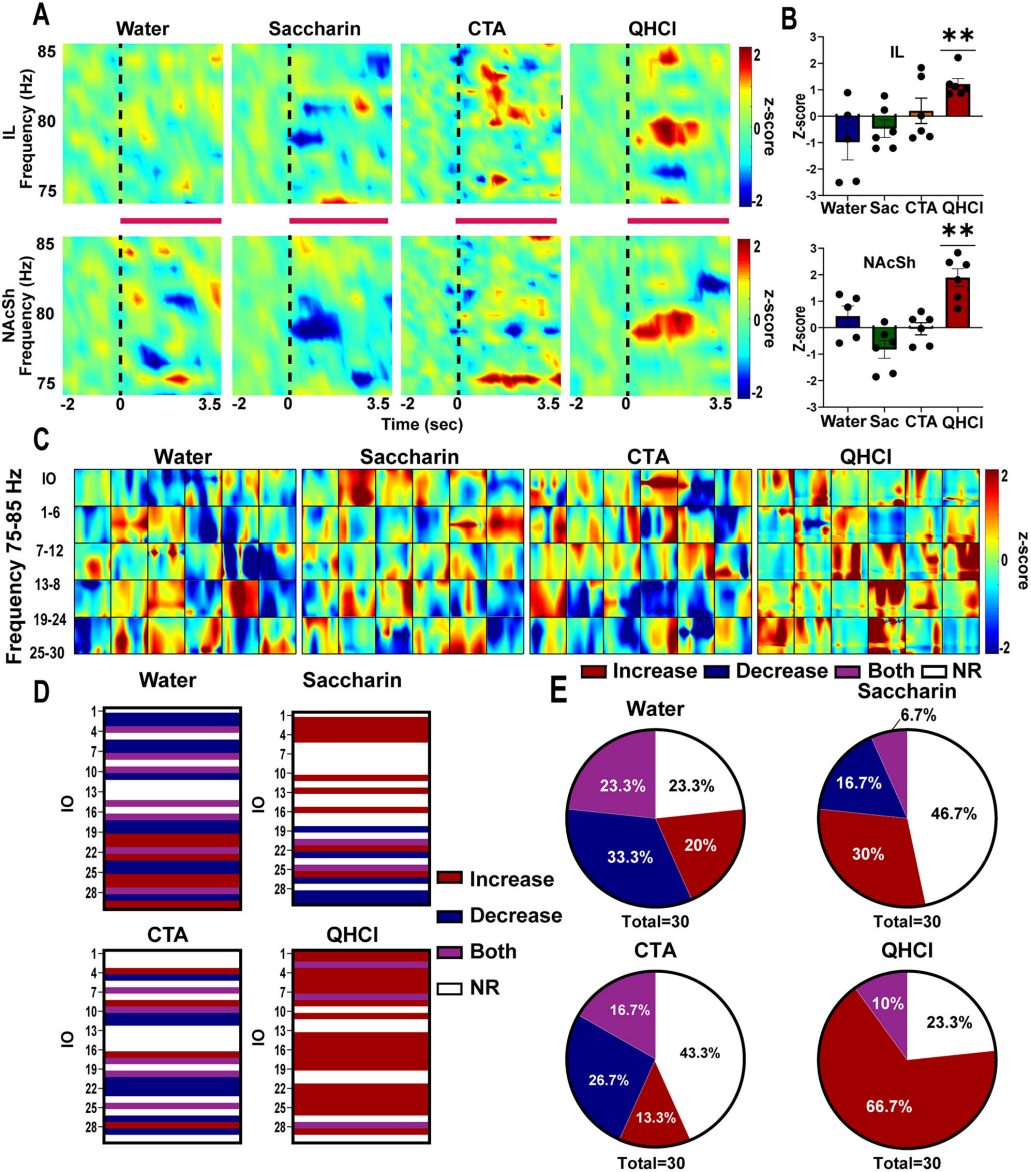
High gamma activity in the IL and NAcSh is involved in regulating innate but not learned aversion in female rats. ***A***, Average perievent spectrograms (*z*-score) showing changes in oscillatory activity after IO infusions (onset of infusion represented by dotted black line) during water (left), saccharin (middle-left), CTA (middle-right), and quinine (right) in both the IL (top) and NAcSh (bottom). *Z*-score relative to baseline is shown on overlay (right). ***B***, Bar graphs showing the mean *z*-score at 80 Hz for each rat during water (blue), saccharin (Sac -green), CTA (orange), and quinine (red) in the IL (top) and the NAcSh (bottom). ***C***, Average coherograms (*z*-score) during 3.5 s infusion duration in response to each of the 30 infusions during water (left), saccharin (middle-left), CTA (middle-right), and quinine (right). *Z*-score relative to baseline is shown on overlay. *z* ≥ 2 were considered increase (red) in coherence, *z* ≤ 2 were considered a decrease (blue) in coherence, and −2 ≤ *z* ≤ 2 was considered no change in coherence. ***D***, Categorization of each infusion (IO) of water (top left), saccharin (top right), CTA (bottom left), and quinine (bottom right) according to its coherence response. Each infusion elicited either an increase (red), decrease (blue), both (purple), or no change (NC-white) in coherence. ***E***, Pie charts showing the percentage of infusions that elicited an increase (red), decrease (blue), both (purple), or no change (NC-white) in coherence for water (top left), saccharin (top right), CTA (bottom left), and quinine (bottom right). QHCl, quinine; CTA, conditioned taste aversion; IL, infralimbic cortex; NAcSh, nucleus accumbens shell. **p < 0.01.

Next, we used LFP recordings to examine how high gamma signaling within the IL→NAcSh pathway processes reward and aversion in female rats. As for male rats, time–frequency analyses of LFP activity were used to assess how oscillatory activity at high gamma frequency in the IL and the NAcSh of female rats varies in response to taste stimuli of different hedonic valences. Figure 3*A* shows spectrograms depicting changes in LFP oscillatory activity in response to water (left), saccharin (middle-left), saccharin following CTA (middle-right), and QHCl (right). Unlike males, the neutral stimulus (water) did not produce changes in activity. When the taste stimulus was salient, results were more variable. For example, while saccharin tended to reduce oscillatory activity in both the IL and NAcSh, CTA appeared to produce an activation, but outside the 80 Hz range. Interestingly, QHCl was the only tastant that produced a consistent increase in oscillatory activity in both the IL and the NAcSh ∼80 Hz. Bar graphs in Figure 3*B* summarize average activity for each group within the IL (top) and NAcSh (bottom). Here, only QHCl produced a significant change from baseline in both the IL (water: *t*_(4)_ = 1,462, *p* = 0.2176; saccharin: *t*_(5)_ = 1.434, *p* = 0.2111; CTA: *t*_(5)_ = 0.4161, *p* = 0.6946; QHCl: *t*_(5)_ = 5.843, *p* = 0.0021) and the NAcSh (water: *t*_(4)_ = 1,145, *p* = 0.316; saccharin: *t*_(5)_ = 2.453, *p* = 0.0577; CTA: *t*_(5)_ = 0.176, *p* = 0.867; QHCl: *t*_(5)_ = 5.648, *p* = 0.0024).

Next, as for males, we assessed how the taste stimuli affect IL→NAcSh functional connectivity at the high gamma frequency band in females by analyzing changes in coherence during the IO infusions. Figure 3*C* shows average coherograms (change in coherence relative to baseline across time) in response to each of the 30 IO infusions of water (left), saccharin (middle-left), saccharin following CTA (middle-right), and QHCl (right). Figure 3*D* categorizes these findings by determining whether each infusion produced an increase (red; *z* ≥ 2), a reduction (blue; *z*−≤2), simultaneous increase and decrease (purple), or no change (white) in coherence across tastants. Here, we observed that, in females, both water and saccharin produced an almost equal distribution of sessions, while most of the trials in CTA did not show a change in coherence. Interestingly, QHCl produced a consistent increase in IL→NAcSh functional connectivity across the majority of infusions. Figure 3*E* shows pie charts quantifying the proportion of responses across tastants. In females, when the taste stimulus was neutral (i.e., water), 33.3% showed a reduction, 23.3% showed both, 23.3% showed no change in coherence, and 20% elicited an increase in IL→NAcSh coherence. When the taste stimulus was rewarding (saccharin), 46.7% of the infusions elicited no change in coherence, 6.7% showed a simultaneous increase and decrease, 30% showed an increase, and 16.7% showed a reduction in coherence. CTA produced 43.3% of infusions as nonresponsive, 26.7% as reduced coherence, and a small proportion showing both (16.7%) or an increase (13.3%) in coherence. As such, no significant differences in the distribution of coherence were observed between water and saccharin (χ^2^_(2,60)_ = 7.378, *p* = 0.0608), water and CTA (χ^2^_(2,60)_ = 2.756, *p* = 0.4309), or saccharin and CTA (χ^2^_(2,60)_ = 3.938, *p* = 0.2682). In contrast, QHCl predominantly elicited an increase in coherence (66.7%) with fewer infusions eliciting no change (23.3%) and only 10% showing both an increase and decrease. No quinine infusions produced a decrease in coherence. Consequently, the coherence profile elicited by quinine was significantly different from water (χ^2^_(2, 60)_ = 19.14, *p* = 0.0003), saccharin (χ^2^_(2, 60)_ = 17.81; *p* = 0.0005), and CTA (χ^2^_(2,60)_ = 21.49, *p* < 0.0001). Collectively, these results show that quinine produced an activation at 80 Hz in both the IL and NAcSh that was associated with an increase in IL→NAcSh functional connectivity, supporting an important role of the IL→NAcSh pathway in encoding innate aversion in female rats. Since we only observed significant activity in females in the high gamma frequency range during innate aversion, the remainder of our investigation focused solely on female rats.

### High gamma LFP lag between the IL and NAcSh is associated with aversive responses elicited by quinine in female rats

**Figure 4.**
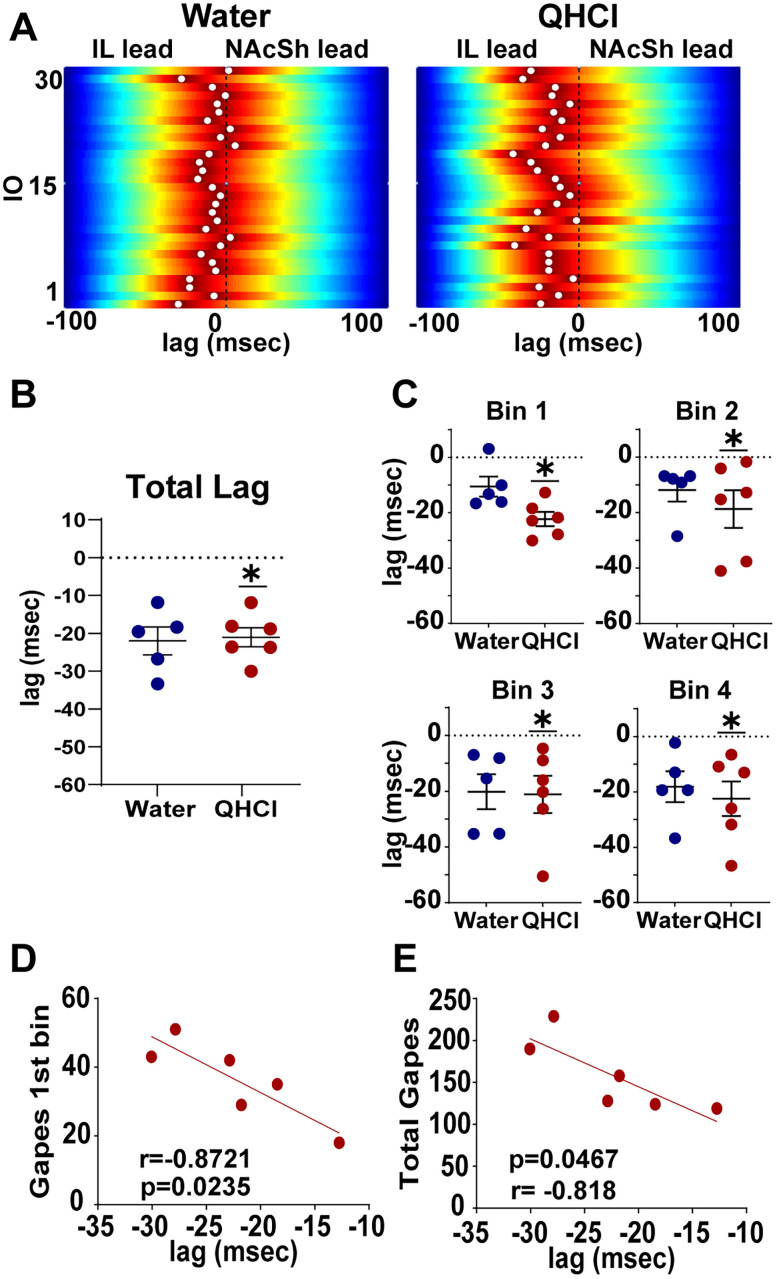
High gamma IL→NAcSh lag is associated with aversive responses elicited by quinine in female rats. ***A***, Examples of normalized color plots of amplitude cross-correlations for each of the 30 infusions (IO) of water (left) and QHCl (right). Warmer colors represent higher cross-correlation. Maximum cross-correlation values are represented by the white dot. Negative lags indicate IL lead and positive lags indicate NAcSh lead. Zero lag indicates no lead. ***B***, Quantification of the average lag for each rat during water (blue) and quinine (red) sessions. ***C***, Average lag for each rat during each of the four bins of water (blue) and quinine (red) sessions. Bin 1, IOs 1–7; Bin 2, IOs 8–14; Bin 3, IOs 15–22; Bin 4, IOs 23–30. ***D***, Correlation between IL→NAcSh lag during Bin 1 and gapes during Bin 1 of the quinine session. ***E***, Correlation between IL→NAcSh lag during Bin 1 and total gapes of the quinine session. *r* = Pearson's correlation coefficient. *p* = *p* value. *p < 0.05.

The above findings show that, in female rats, quinine infusion increased oscillatory activity in both the IL and NAcSh as well as IL→NAcSh coherence, suggesting that these regions are activated and work as a circuit to perhaps drive the expression of innate aversive behaviors and optogenetic stimulation of this pathway alters innate but not learned aversion. To examine this finding further, we analyzed the directionality of information using power-power cross-correlation between the IL and NAcSh LFP signals. We compared the temporal relationship of IL and NAcSh signals between innate aversive (i.e., QHCl) and neutral (i.e., water) taste stimuli. Figure 4*A* shows examples of the power-power cross-correlation across each of the 30 IO infusions for water (left) and QHCl (right) sessions; the maximum cross-correlation value is noted with a white dot. A negative lag indicates that the IL leads over the NAcSh, a positive lag indicates the NAcSh leads over the IL, while a lag = 0 indicate no clear lead. In these examples we observe that, while cross-correlation tends to reach its peak around zero during the infusion of water, during most of the quinine infusions, the cross-correlation peak is negative, indicating a lead of the IL signal over the NAcSh. Figure 4*B* quantifies average lag for each rat during both water (blue) and quinine (red) sessions; quinine was observed to be significantly different from zero (Wilcoxon signed-rank test, *p* = 0.0312) while water was not (Wilcoxon signed-rank test, *p* = 0.0625). Figure 4*C* quantifies lags across infusions separated into four bins for each rat in response to water and quinine to investigate how lag changes across the session. In the first bin (IOs 1–7), there is no clear lead during the infusion of water (Wilcoxon signed-rank test, *p* = 0.125). Interestingly, during the infusion of quinine in Bin 1, there was a clear IL lead that was significantly different (Wilcoxon signed-rank test, *p* = 0.0312) from 0 (no lead). In subsequent bins (Bin 2: IOs 8–14; Bin 3: IOs 15–22; Bin 4: IOs 23–30), lag was consistently significantly different from 0 during quinine infusion (Bin 2 *p* = 0.0312, Bin 3 *p* = 0.0312, Bin 4 *p* = 0.0312) but not during water (Bin 2 *p* = 0.0625, Bin 3 *p* = 0.0625, Bin 4 *p* = 0.0625). Finally, to assess whether the lag within each bin correlates with the aversive responses during the corresponding bin, as well as with subsequent aversive responses later in the session, we correlated lags within each bin to aversive responses during that bin and with total aversive responses during the session. Interestingly, a significant negative correlation between lags and gapes was observed only during Bin 1 (IOs 1–7; *r* = −0.881, *p* = 0.0205; Fig. 4*D*) and between Bin 1 lags and total aversive responses during the session (*r* = −0.818, *p* = 0.0467; Fig. 4*E*). These findings indicate that the greater the lag between the IL and NAcSh early in the session, the more aversive responses rats elicited during that first bin; furthermore, a higher lag in the first bin can predict an increase in aversive responses throughout the entire quinine session. These findings suggest that the IL→NAcSh lag not only modulates immediate aversive behaviors within the initial time frame but also predicts sustained aversive responding across the session duration.

### High gamma optical stimulation of the IL-NAcSh pathway altered innate but not learned aversion in female rats

**Figure 5.**
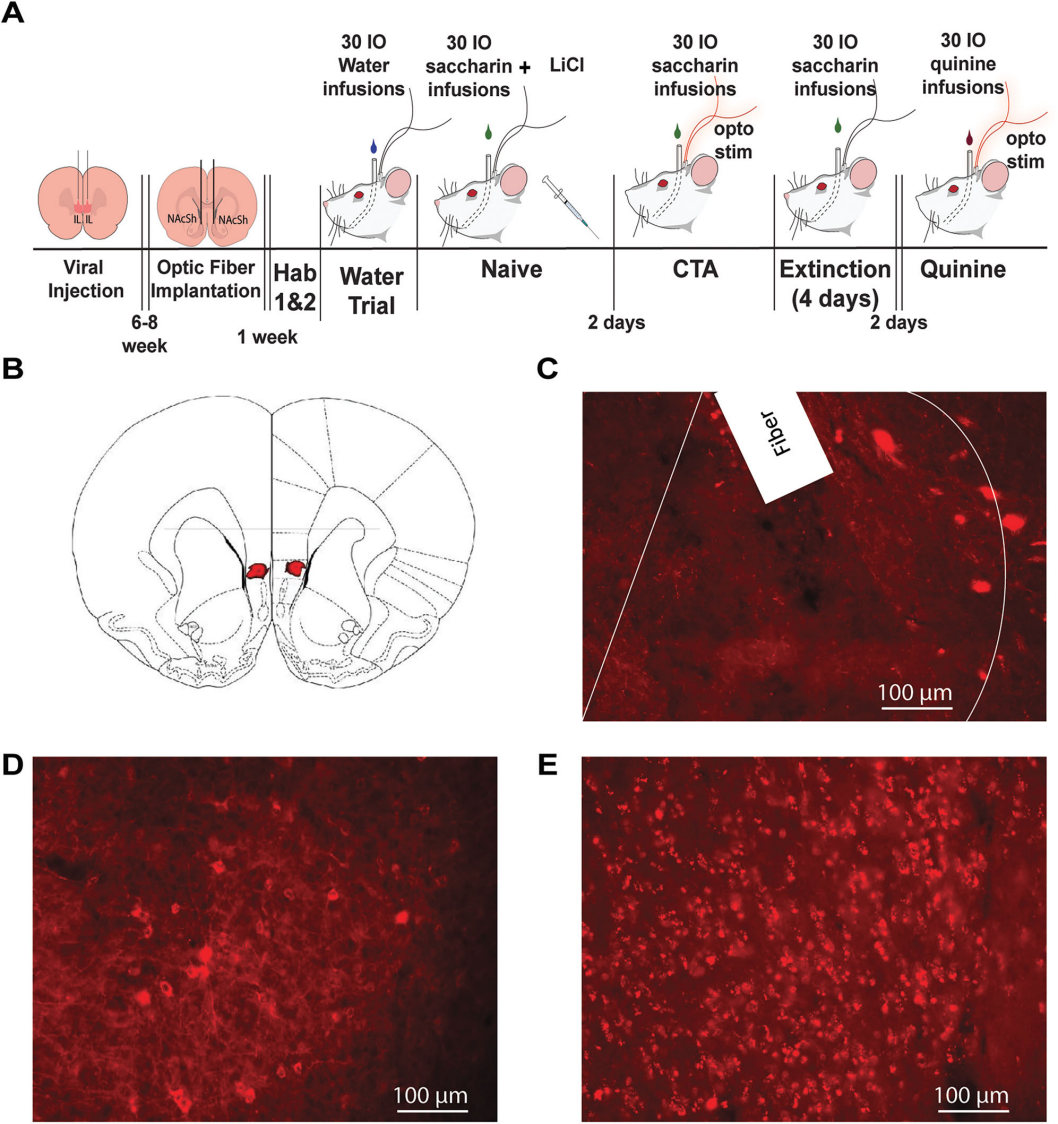
Optogenetic experiment: schematic timeline and histological verification of viral expression. ***A***, Schematic timeline: rats received virus microinjections with 6 weeks for virus expression. Optical fibers and IO cannulas were implanted and following 1 week recovery rats were habituated to IO water infusions. Rats then received IO infusions of saccharin followed by an injection of LiCl to create an association between saccharin and illness and then given a 2 d break before CTA testing. During the CTA session, each IO saccharin infusion was paired with 80 Hz optical activation of the IL–NAcSh pathway. This was followed by 4 d of saccharin only IO infusions (extinction), followed by a final day in which IO infusions of quinine was paired with 80 Hz optogenetic stimulation of the IL–NAcSh pathway. ***B***, Wide-field image of viral expression in the IL overlaid onto an atlas plane and (***C***) expression of NAcSh projections (the solid line delineates NAcSh) and optical fiber. Image of neuron cell body viral expression in the IL (***D***) and in the NAcSh projections (***E***).

To assess causality of high gamma activity in the IL and NAcSh on innate but not learned aversion in female rats, we investigated if high gamma (80 Hz) optical stimulation of the IL→NAcSh pathway had any effect on learned or innate aversion in female rats (Fig. 5*A*). Viral expression was histologically verified in the IL and NAcSh projections, as illustrated in Figure 5. Figure 5*B* shows viral expression in the IL overlaid onto an atlas plane. Optical fiber placement and viral expression in NAcSh projections are shown in Figure 5*C* with the borders of the NAcSh represented by the solid white lines. Neural cell body viral expression is shown in the wide-field images for both the IL (Fig. 5*D*) and the NAcSh (Fig. 5*E*).

**Figure 6.**
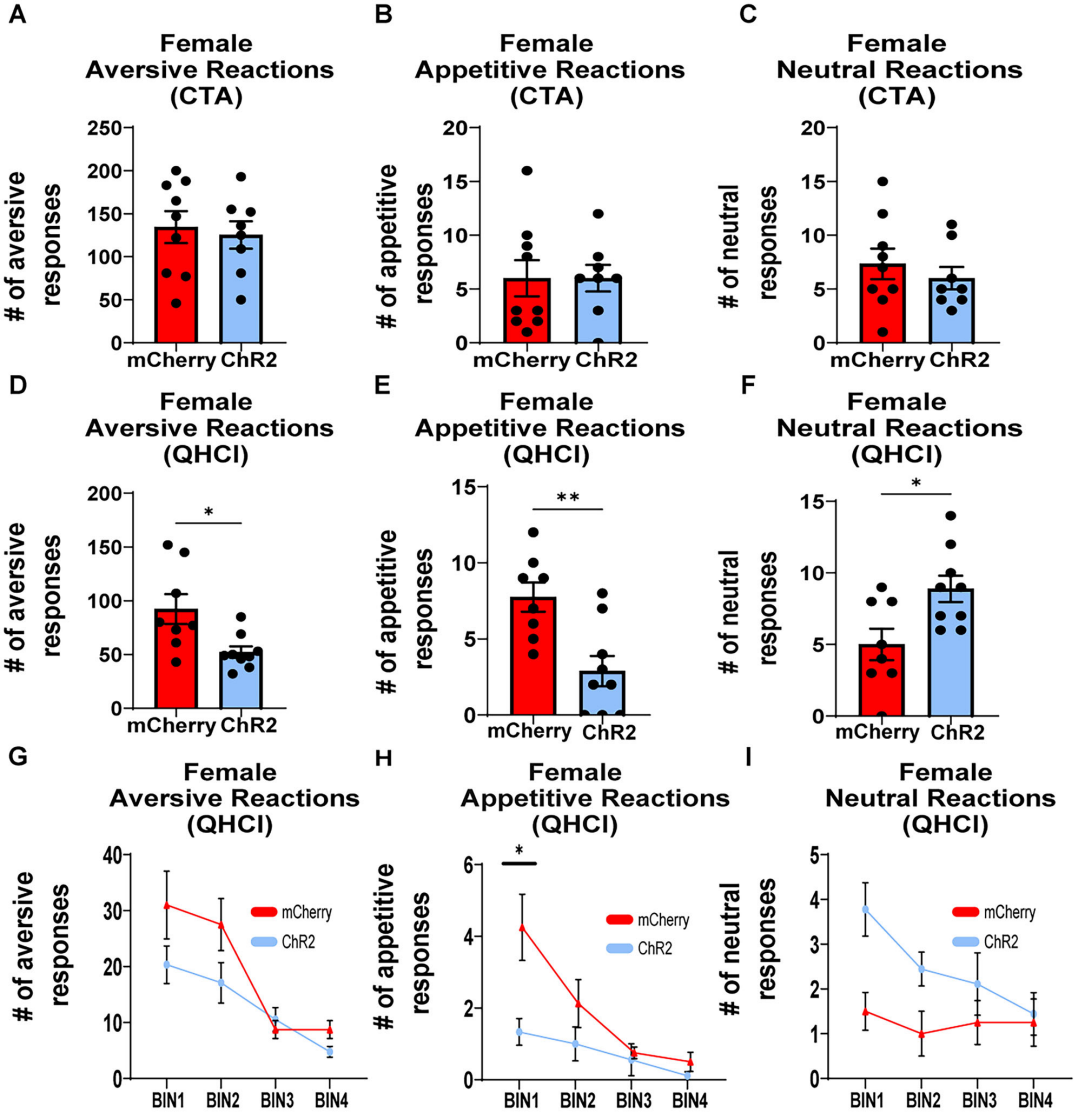
80 Hz stimulation of the IL→NAcSh pathway alters innate but not learned (CTA) aversion in female rats. Top: Bar graphs shows the mean number of aversive (***A***), appetitive (***B***), and neutral (***C***) responses during the CTA sessions. Middle: Bar graphs show the mean number of aversive (***D***) appetitive (***E***) and neutral (***F***) responses during the quinine (QHCl) sessions. Bottom: Line graphs show the mean ± SEM aversive (***G***), appetitive (***H***), and neutral (***I***) responses across each 5 min bin. CTA, conditioned taste aversion; QHCL, quinine; ChR2, channelrhodopsin. *p < 0.05, **p < 0.01.

Interestingly, optical stimulation did not have an effect on either aversive (*t*_(8,7)_ = 0.3768, *p* = 0.7116; Fig. 6*A*), appetitive (*t*_(8,7)_ = 0, *p* > 0.99; Fig. 6*B*), or neutral (*t*_(8,7)_ = 0.7574, *p* = 0.4612; Fig. 6*C*) TR during learned aversion (CTA). In contrast, 80 Hz optical stimulation significantly decreased both aversive (*t*_(7,8)_ = 2.700, *p* = 0.0243; Fig. 6*D*) and appetitive (*t*_(8,7)_ = 3.523, *p* = 0.0031; Fig. 6*E*) TR during quinine infusions. To ensure that stimulation was not simply reducing overall responding, we analyzed neutral reactions and found that this same stimulation produced an increase in neutral responses to quinine infusions (*t*_(7,8)_ = 2.710; *p* = 0.0168; Fig. 6*F*) indicating that stimulation is causing the response profile to the quinine infusions to shift from an aversive state to a neutral state. Next, we examined whether optical stimulation effected the natural habituation to the quinine infusions. To do this, we divided the session into four bins and examined the time course of these responses during the quinine infusions. For aversive TR (Fig. 5*G*), a two-way ANOVA revealed a significant effect of bin (*F*_(3,45)_ = 17.06, *p* < 0.0001) and treatment (*F*_(3,45)_ = 4.641, *p* = 0.0479), but no bin × treatment interaction (*F*_(3,45)_ = 1.746, *p* = 0.1711). However, optical stimulation reduced appetitive TR specifically during the first bin (Fig. 6*H*). This was confirmed by a two-way ANOVA revealing a significant main effect of treatment (*F*_(1,15)_ = 10.63, *p* = 0.0053), bin (*F*_(2.180,32.69)_ = 10.80, *p* < 0.001), and a bin × treatment interaction (*F*_(3,45)_ = 3.369, *p* = 0.0266) and confirmed by post hoc analysis (*p* = 0.0161). Finally, a two-way repeated-measures ANOVA for neutral reactions (Fig. 6*I*) revealed only a significant effect of treatment (*F*_(1,15)_ = 12.55, *p* = 0.0030) but no significant effect of bin (*F*_(3,45)_ = 2.107, *p* = 0.1127) or bin × treatment interaction (*F*_(3,45)_ = 1.343, *p* = 0.2722). Collectively, these findings indicate that high gamma optical activation of the IL→NAcSh pathway specifically alters innate (quinine) but not learned (CTA) aversion in female rats, particularly during initial IO infusions of quinine.

## Discussion

An impaired ability to modulate aversive behaviors is prevalent in several psychiatric disorders such as depression and SUDs (Rzepa et al., 2017; McHugh and Kneeland, 2019; Liang et al., 2022). Given the importance of the IL→NAcSh pathway in affect regulation and the involvement of high gamma frequencies in reward/aversion processing (Masimore et al., 2005; Van Der Meer, 2009; Donnelly et al., 2014; West et al., 2021), here we investigated the role of 80 Hz high gamma frequency in the IL→NAcSh circuit in innate and learned aversion across sex. Using LFP electrophysiology, clear sex differences were observed in the role of the IL and NAcSh and their coherence in innate and learned aversion. Specifically, in males, while 80 Hz high gamma signaling in the IL→NAcSh pathway was not involved in learned or innate aversion, this pathway exhibited higher synchronization (functional connectivity) while processing a neutral taste (water) stimulus. Conversely, in females, the IL and NAcSh both exhibit stronger oscillatory activity and clear top-down directionality when processing the innate aversive stimulus. Moreover, 80 Hz optical stimulation of the IL→NAcSh pathway in females further confirmed the involvement of this frequency band as it reduced innate but not learned aversion. Collectively, these results indicate that at the high gamma frequency range, the ILNAcSh pathway plays differential roles in affective processing across sex, modulating neutral or ingestive behaviors in males, and innate aversion in female rats. Each of these results is discussed in detail below.

First, we explored the role of IL→NAcSh pathway at the high gamma frequency range in male rats during reward, aversion, and neutral stimuli using LFP recordings. Analysis revealed that both the IL and NAcSh showed higher synchronized activity during the infusion of a neutral water stimulus, but not during the infusion of salient (appetitive or aversive) tastants. These results indicate that, at the high gamma frequency range, the IL→NAcSh circuit may not play a role in modulating learned or innate aversion but may be involved in modulating neutral or ingestive behaviors in males. This is consistent with literature showing that gamma oscillations regulate feeding behaviors in rodent models (Carus-Cadavieco et al., 2017; Dwiel et al., 2019). For example, LFP gamma activity in the NAcSh of male rats follows a predictable pattern with increased gamma activity just prior to feeding onset, stabilizing during feeding, and then reversing to non-feeding levels after feeding was terminated (Dwiel et al., 2019). Overall, these results suggest that high gamma activity in the IL→NAcSh of male rats may be associated with neutral feeding behavior and not with the expression of salient hedonic behaviors, innate or learned. Since we did not observe any significant results during learned or innate aversion in males, the remainder of this report focused on female rats.

Next, we explored the role of the IL→NAcSh pathway at the high gamma frequency range in female rats during reward, aversion, and neutral stimuli using LFP recordings. First, we found that only quinine infusions produced an increase in oscillatory activity in both the IL and the NAcSh at 80 Hz, with an increase in IL→NAcSh coherence observed during most of the quinine infusions, consistent with literature showing that gamma oscillations are associated with aversion (Dejean et al., 2016; Tan et al., 2019). Interestingly, while CTA appeared to produce increased activity in the IL and NAcSh, it was mostly uncoordinated activity and outside the 80 Hz range. As such, while high gamma frequencies may be involved in aversion in general, evidenced by an increased activity at different frequencies within this band during both quinine and CTA sessions, it is the coordinated activity at 80 Hz that appears to be involved in modulating innate aversion in females.

Based on these observations, we aimed to characterize the signaling dynamics of the IL→NAcSh pathway during quinine infusions to elucidate a mechanism by which this circuit encodes innate aversion. To do this, we investigated the directionality between the IL and the NAcSh during quinine-induced aversion compared with a neutral water stimulus. We found that during the infusion of quinine, there was a significant IL lead that was not observed during infusion of the neutral water stimulus, suggesting a possible top-down directionality during the processing of innate aversion. Next, to examine if this possible top-down directionality changed over the course of the session, we segmented the session into four equal bins and analyzed the lag within each specific bin. Results demonstrated that during quinine infusions, lag was consistently different from zero across the entire session, further indicating a consistent top-down directionality during quinine infusions.

To investigate how this lag may modulate behavior, we analyzed the correlation between the lag within each bin and the aversive responses observed within the corresponding bin. The observed lag in the first bin correlated with the expression of aversive behaviors during that first bin. These results indicate that, initially, this top-down directionality may drive aversive responding to quinine infusions. Next, to examine if lag within each bin exerted a sustained effect on aversive responding or solely drove initial responding, we correlated the lag within each bin with the number of aversive reactions across the entire session. Here, we found that the lag in the first bin correlated with the expression of aversive behaviors during the entirety of the session. This indicates that in addition to driving initial responding, initial lag is also predictive of overall responses across the session. Collectively, these findings indicate that high gamma activity within the IL→NAcSh pathway may function to drive the initiation and sustained response to innately aversive stimuli in females.

Given these findings, we next examined a possible causal link of the IL→NAcSh circuit in modulating learned and innate aversion in females using optogenetics. We found that 80 Hz optical stimulation of this pathway altered innate but not learned aversion, causing a decrease in both appetitive and aversive reactions and an increase in neutral reactions to quinine. Interestingly, our results show that high gamma stimulation of the IL→NAcSh circuit did not abolish orofacial responses; instead, stimulation shifted affective behavioral responses to quinine infusions from an aversive to neutral profile. Of note, previous findings from our lab showed that 20 Hz IL→NAcSh pathway optical stimulation only reduced CTA in male rats but did not have an effect on quinine aversion in either male or female rats (Hurley et al., 2020). The current findings build upon that work and demonstrate that the modulation of different types of aversion (innate vs learned) via the IL→NAcSh pathway is both sex and frequency specific.

Given our LFP data showing a top-down directionality, one would predict that activation of this circuit during quinine infusions using 80 Hz optical stimulation would produce an increase in gaping behavior. However, our optogenetic studies showed the opposite; 80 Hz stimulation of the IL→NAcSh circuit during quinine infusions elicited a reduction in both appetitive and aversive behaviors and an increase in neutral behaviors. Therefore, it is possible that enhancing the normal activation elicited by quinine with 80 Hz optical stimulation may, in fact, overstimulate the NAcSh and, by doing so, block the afferent information from the IL involved in modulating aversive responding.

Additionally, 80 Hz optical overstimulation of NAcSh may also inhibit the flow of efferent information onto downstream targets associated with the expression of aversion, such as the lateral hypothalamus (LH; Usuda et al., 1998; Heimer et al., 1991; He et al., 2023). Notably, the NAcSh-LH pathway is involved in regulating aversive behaviors (Li et al., 2013; De Jong et al., 2018; He et al., 2023). For example, optical stimulation of the NAcSh to LH circuit increased interaction with an aversive stimulus during an approach-avoidance task, while optical inhibition decreased interaction time (He et al., 2023). Future studies are needed to investigate whether overstimulating IL terminals in the NAcSh inhibits its output to the LH, thereby decreasing aversive reactions similarly to direct stimulation of the NAcSh-LH pathway and investigate if this is consistent across sex. Likewise, future research could further extend our findings by investigating oscillatory activity in additional brain regions involved in negative hedonic processing, such as the basolateral amygdala (BLA) or anterior insular cortex (aIC) which project to the NAcSh (Wright et al., 1996; Di Ciano and Everitt, 2004; Gehrlach et al., 2020).

### Concluding remarks

The current findings are consistent with and build upon previous work showing that high gamma frequencies are involved in various forms of aversion processing (Dejean et al., 2016; Tan et al., 2019). Importantly, however, our results also show clear sex differences in how innate as well as learned aversion are encoded and modulated in the IL→NAcSh circuit. While the literature investigating innate aversion modulation is limited, and most of the studies incorporate only male rats, understanding how innate aversion differs across sex should add valuable insight into effective treatment strategies for psychiatric illnesses. For example, our findings support the contention that the IL→NAcSh pathway may be a possible target for disorders in which negative hedonic processing is dysregulated, such as obsessive-compulsive disorder, PTSD, anxiety, and eating disorders (Davey, 2011; Dunsmoor and Paz, 2015; Anderson et al., 2021; Tolchinsky et al., 2024). Indeed, high gamma stimulation has been used in animal models to improve obsessive-compulsive behaviors (McCracken and Grace, 2007) and anxiety (Lim et al., 2015). However, in humans, “high” frequency stimulation using either transcranial magnetic stimulation or transcranial alternating current stimulation have been found to improve obsessive-compulsive behaviors (Grover et al., 2021), PTSD (Cohen et al., 2004; Boggio, 2024), and eating disorders (Baczynski et al., 2014; Muratore et al., 2021) but often involve stimulations <30 Hz. While 80 Hz is considered too rapid for human neural stimulation and cannot be directly translated from animal studies to humans, our data emphasizes that the neural circuits involved in modulating these behaviors that are disrupted during illness are frequency specific and sex specific, suggesting the need for a more nuanced approach to study these topics.

## Synthesis

Reviewing Editor: Mihaela Iordanova, Concordia University - Loyola Campus Decisions are customarily a result of the Reviewing Editor and the peer reviewers coming together and discussing their recommendations until a consensus is reached. When revisions are invited, a fact-based synthesis statement explaining their decision and outlining what is needed to prepare a revision will be listed below. The following reviewer(s) agreed to reveal their identity: NONE. Note: If this manuscript was transferred from JNeurosci and a decision was made to accept the manuscript without peer review, a brief statement to this effect will instead be what is listed below.

The authors have addressed the comments of the reviewers.

A couple of minor comments that authors may want address during the proofs stage:

1) TR could be spelled out. It is to a standard abbreviation and reduced readability of the Ms.

2) line 566 - 'in'

3) Fig 4D and E - location of 'p' and 'r' are inconsistent across panels

4) Fig 5 A - text under the horizontal timeline is not levelled.
